# GBP5 drives malignancy of glioblastoma via the Src/ERK1/2/MMP3 pathway

**DOI:** 10.1038/s41419-021-03492-3

**Published:** 2021-02-19

**Authors:** Xiaoting Yu, Jing Jin, Yanwen Zheng, Hua Zhu, Hui Xu, Jun Ma, Qing Lan, Zhixiang Zhuang, Clark C. Chen, Ming Li

**Affiliations:** 1 Central Laboratory, The Second Affiliated Hospital of Soochow University, Suzhou, Jiangsu Province China; 2 Department of Oncology, The Second Affiliated Hospital of Soochow University, Suzhou, Jiangsu Province China; 3 Department of Neurosurgery, The Second Affiliated Hospital of Soochow University, Suzhou, Jiangsu Province China; 4 Department of Pediatrics, The First Hospital of China Medical University, Shenyang, China; 5 Department of Neurosurgery, University of Minnesota, Minneapolis, MN USA

**Keywords:** CNS cancer, Oncogenes

## Abstract

Guanylate binding proteins (GBPs), a family of interferon-inducible large GTPase, play a pivotal role in cell-autonomous immunity and tumor malignant transformation. Glioblastoma (GBM) is the most prevalent and lethal primary brain tumor in adults. Here we show that GBP5 was highly expressed in GBM cell lines and in clinical samples, especially in the mesenchymal subtype. The expression levels of GBP5 were negatively correlated with the prognosis of GBM patients. Overexpression of GBP5 promoted the proliferation, migration, and invasion of GBM cells in vitro and in vivo. In contrast, silencing GBP5 by RNA interference exhibited the opposite effects. Consequently, targeting GBP5 in GBM cells resulted in impaired tumor growth and prolonged survival time of mice with GBM tumors. We further identified that the Src/ERK1/2/MMP3 axis was essential for GBP5-promoted GBM aggressiveness. These findings suggest that GBP5 may represent a novel target for GBM intervention.

## Introduction

Gliomas, the most common and lethal primary intracranial tumors in adults, originate from de-differentiated glial cells or glial-like precursors^1^. Glioblastoma (GBM) is the most aggressive subtype of glioma, accounting for more than half of all astrocytoma cases, which has been categorized as a World Health Organization grade IV glioma. GBM is a heterogeneous tumor characterized by angiogenesis, proliferation, invasion, and evasion of apoptosis. Despite radical treatment, recurrence rate of GBM remains as high as 90%. Patients with GBM have a median overall survival of 15–18 months with treatment, and the 5-year survival is <10%^2^.

Human guanylate binding protein 5 (GBP5) belongs to the dynamin superfamily of interferon-gamma-inducible large GTPases^3^, which are considered as central orchestrators of neoplastic diseases immunity^4^. GBP5 has three splicing variants (GBP5a, 5b, and 5ta) and forms two different proteins (GBP5a/b and GBP5ta), both of which are expressed in a restricted pattern. The restricted expression pattern and the pivotal role of some well-known members of the GBP family in proliferation and invasion suggest potential malignancy-related functions of GBP5^5^. As reported, GBP5 is expressed highly in gastric adenocarcinomas and medullary carcinoma^6,7^. In addition, GBP5 is positively correlated with PD-L1 expression in human glioma, endowing it the role of a complementary prognostic indicator for anti-PD-1/PD-L1 therapy^8^. However, its function in GBM remains elusive.

In current studies, we examined the expression profile of GBP5 in GBM cell lines and in clinical specimens and investigated its biological function both in vitro and in vivo. The underlying molecular mechanisms were also explored. It’s found that GBP5 is highly elevated in GBMs and its expression promoted GBM malignancy, including cell migration, invasion, and proliferation. Moreover, we showed that the SRC/ERK1/2/MMP3 pathway was essential for GBP5-mediated GBM aggressiveness.

## Results

### GBP5 is upregulated in GBM tumors and cell lines and predicts poor outcomes

**Fig. 1:**
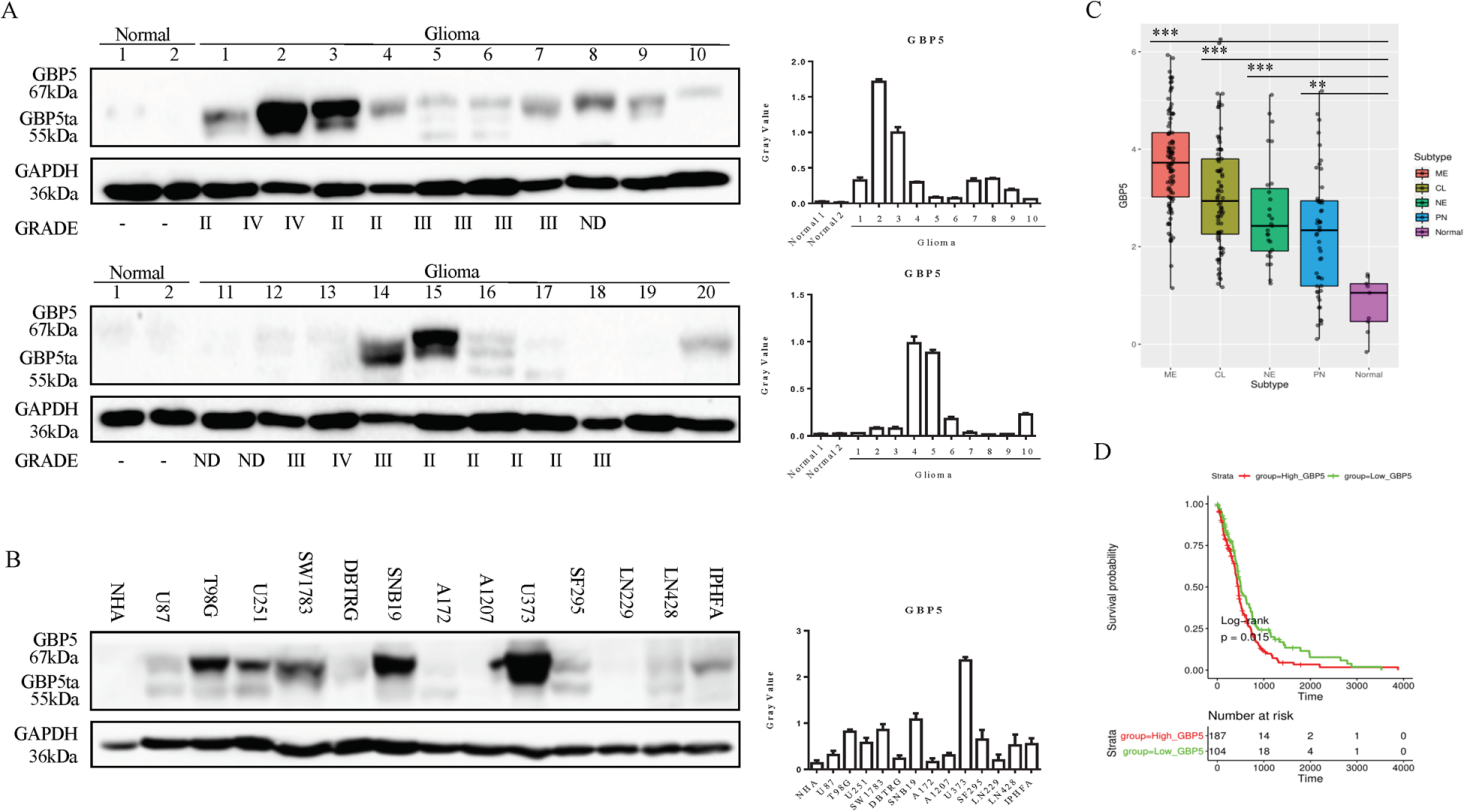
GBP5 is upregulated in GBM tumors and cell lines and predicts poor prognosis. **A** Immunoblot analysis of GBP5 in human GBM patient samples and normal brain tissues. GAPDH was used as loading control. **B** Immunoblot analysis of GBP5 in human glioma cell lines, immortalized normal astrocyte NHA, and immortalized primary human fetal astrocyte IPHFA cells. GAPDH was used as loading control. **C** Cancer Genome Atlas (TCGA) dataset analysis of the GBP5 mRNA expression levels in GBM subtypes and normal brain tissues. **p < 0.01; ***p < 0.001. Normal: *n* = 9; Neural (NE): *n* = 27; classical (CL): *n* = 80; mesenchymal (ME): *n* = 96. **D** TCGA dataset analysis of the relationship between the expression levels of GBP5 and the prognosis of GBM patients. *p* = 0.015. *n* = 331.

We first examined the expression profile of GBP5 in 20 GBM patient samples by western blot. These GBM tumors included 7 Grade II, 7 Grade III, and 3 Grade IV samples. The results showed that the adjacent normal brain tissues expressed low levels of GBP5, while 14 of 20 (70%) tumor samples expressed higher levels (Fig. 1A). We noticed that 8 of the 14 tumor samples expressed high levels of full-length GBP5a/b, while 6 of 14 tumor samples expressed the truncated form GBP5ta. Interestingly, the expression levels of GBP5ta were lower than that of GBP5a/b in most of the tumor samples. Moreover, GBP5 was highly elevated in all the Grade IV tumors, and 6 of 7 Grade III tumors, and 3 of 7 Grade II tumors, suggesting GBP5 expression is correlated with tumor grade.

We also examined the protein levels of GBP5 in a collection of 12 glioma cell lines. It was found that the expression levels of GBP5a/b were increased in 5 of 12 (41.67%) cell lines relative to the immortalized normal human astrocyte NHA and primary human fetal astrocyte IPHFA cells (Fig. 1B). Of note, GBP5ta was detected in 2 (T98G and U251) of the five cell lines (T98G, U251, SW1783, SNB19, and U373), and the expression levels of GBP5ta were lower than that of GBP5a/b in these two cell lines. Together with the expression profile of both isoforms in GBM tumors, we speculate that the full-length GBP5a/b might play a more important role in GBM. We, therefore, focused on the full-length form of GBP5a/b (hereafter referred to as GBP5) in the current study. Using the Cancer Genome Atlas (TCGA) dataset to analyze the mRNA expression levels, we found that GBP5 was significantly upregulated in all the four GBM subtypes when compared to the normal brain tissues, and the expression levels of GBP5 in the mesenchymal subtype were the highest (Fig. 1C). Importantly, the expression levels of GBP5 were negatively correlated with the prognosis of the patients (Fig. 1D).

### GBP5 enhances GBM cell proliferation, migration, and invasion

**Fig. 2:**
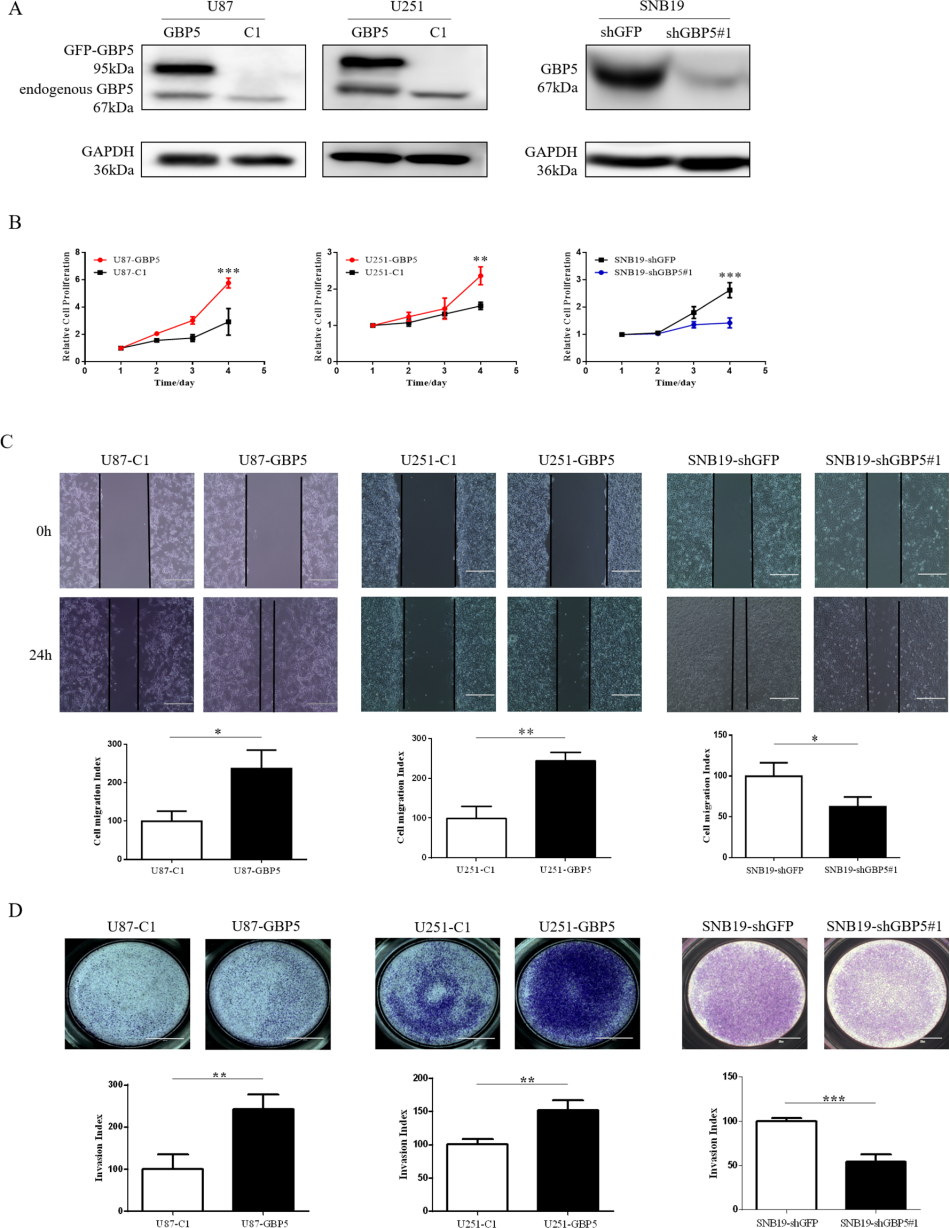
GBP5 promotes GBM cell proliferation, migration, and invasion. **A** Immunoblot analysis of GBP5 in U87-C1, U87-GBP5, U251-C1, U251-GBP5, SNB19shGFP, and SNB19shGBP5 cells. GAPDH was used as loading control. **B** Effect of GBP5 knockdown or overexpression on GBM cell proliferation. **p < 0.01; ***p < 0.001. *n* = 3. **C** Effect of GBP5 on the cell migration assessed by wound healing assay in 24 h. Cell migration index = migrated distance (at 24 h)/scratching distance (at 0 h). *p < 0.05; **p < 0.01. *n* = 3. **D** Effect of GBP5 on the cell invasion by the matrigel transwell assay. Invasion index = OD570 of invaded cells in treatment groups/OD570 of invaded cells in control groups. **p < 0.01; ***p < 0.001. *n* = 3.

To study the biological role of GBP5 in GBM cells, we first did gain-of-function studies using U87 and U251 cell lines, which expressed relatively lower levels of GBP5 (Fig. 1B). The U87 and U251 cells transfected with pEGFP-C1-GBP5 were named U87-GBP5 and U251-GBP5, respectively, while the empty vector-transfected control cells were named U87-C1 and U251-C1. The expression of GBP5 in these two cell lines was verified by Western blot (Fig. 2A). Cell Counting kit-8 (CCK-8) assay was performed to assess the effect of GBP5 expression on cell growth. We found that overexpression of GBP5 significantly enhanced the cell proliferation in both U87 and U251 cells (Fig. 2B). The effect of GBP5 on cell migration was assessed by wound healing assay. GBP5 expression significantly shortened the cell scratches in both U87 and U251 cells at 24 h after cell seeding, suggesting that overexpression of GBP5 could significantly promote the migration ability of GBM cells by more than twofolds (Fig. 2C). The effect of GBP5 on the invasive ability of U87 and U251 cells was detected by the transwell invasion assay. The results showed that overexpression of GBP5 could dramatically enhance the cells’ ability of penetrating matrigel and could promote the invasive ability of U87 and U251 cells by ~2.2 and 1.5 folds, respectively (Fig. 2D).

We used loss-of-function approaches to further verify the results. We chose an established SNB19 cell line, which expressed relatively higher level of GBP5 (Fig. 1B). We found that depletion of GBP5 by lentiviral RNA interference significantly suppressed the proliferation, migration, and invasion of SNB19 cells (Fig. 2A–D). These results suggested that the expression of GBP5 enhanced the proliferation, migration, and invasion of GBM cells.

### MMP3 is essential for GBP5-driven cell proliferation and invasion

**Fig. 3:**
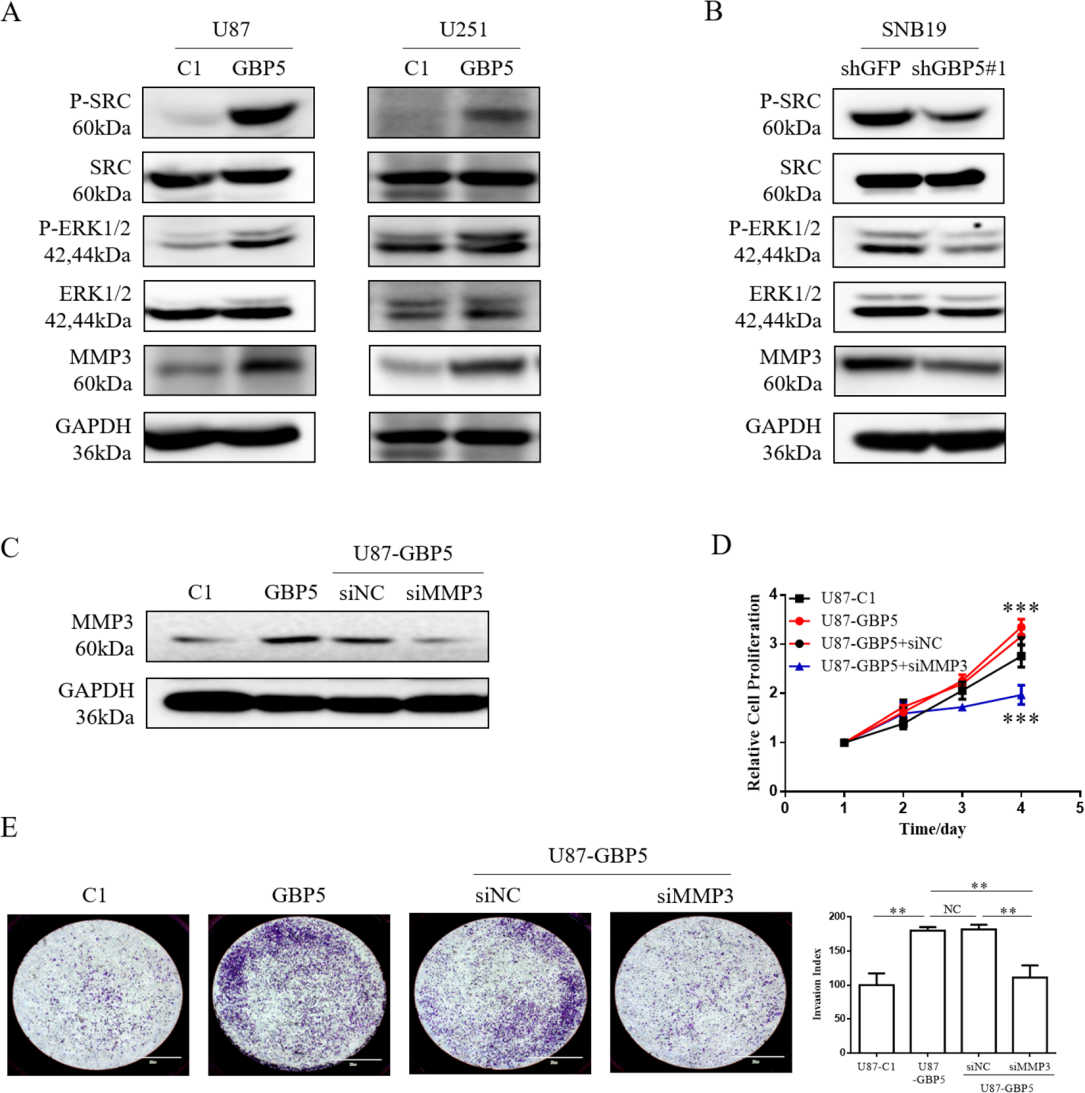
MMP3 is essential for GBP5-driven cell proliferation and invasion. **A** Immunoblot analysis of p-Src, p-ERK1/2, and MMP3 in U87-C1, U87-GBP5, U251-C1, and U251-GBP5 cells. GAPDH was used as loading control. **B** Immunoblot analysis of p-Src, p-ERK1/2, and MMP3 in SNB19shGFP and SNB19shGBP5 cells. GAPDH was used as loading control. **C** Immunoblot analysis of MMP3 in U87-GBP5 cells with or without MMP3 silencing with siRNA. **D** Effect of MMP3 knockdown on the GBP5-driven cell proliferation. ***p < 0.001. *n* = 3. **E** Effect of MMP3 knockdown on the GBP5-driven cell invasiveness by the matrigel transwell assay. Invasion index = OD570 of invaded cells in treatment groups/OD570 of invaded cells in control groups. **p < 0.01. NC = not statistically significant. *n* = 3.

To explore the molecular mechanisms of GBP5-driven GBM cell proliferation and invasion, we screened a variety of metastasis-related genes which may be affected by GBP5 expression in GBM cells by quantitative RT-PCR. These genes included MMP3, MMP14, IL8, FN1, and IL6. We observed that MMP3 expression was significantly induced in both U87-GBP5 and U251-GBP5 cell lines (Supplementary Fig. S1). MMP3 is a zinc-dependent endopeptidase produced by tumor cells and plays an important role in the growth and invasion of the central nervous system tumors^9^. Therefore, we speculated that MMP3 gene may play a role in GBP5-promoted cell proliferation and invasion. The results of quantitative RT-PCR were verified by western blot, which showed that the expression of GBP5-induced higher protein level of MMP3 in U87 and U251 cells (Fig. 3A). Consistent with this, knockdown of GBP5 with siRNA suppressed the expression of MMP3 in SNB19 cells (Fig. 3B).

To determine whether MMP3 plays a role in GBP5-driven GBM cell proliferation and invasion, MMP3 in U87-GBP5 cells was silenced by siRNA. It was found that the depletion of MMP3 aborted the cell proliferation and invasiveness promoted by GBP5 in U87 cells (Fig. 3C, D, E). Therefore, these results suggested that MMP3 was necessary for the GBP5-driven GBM cell proliferation and invasion.

### Src/ERK signaling regulates GBP5-promoted MMP3 expression, cell proliferation and invasion

Next, we sought to identify the signaling pathway essential for GBP5/MMP3-protmoted cell malignancy. Src and ERK have been reported to participate in the MMP3 expression in hepatoma and in osteoarthritis^10–12^, we therefore tested whether GBP5 had influence on the activation of Src and ERK1/2. It was found that in U87 and U251 cells, the expression of GBP5 significantly increased the phosphorylation levels of both Src and ERK1/2, while the silence of GBP5 decreased the levels of p-Src and p-ERK1/2 in SNB19 cells (Fig. 3A, B).

**Fig. 4:**
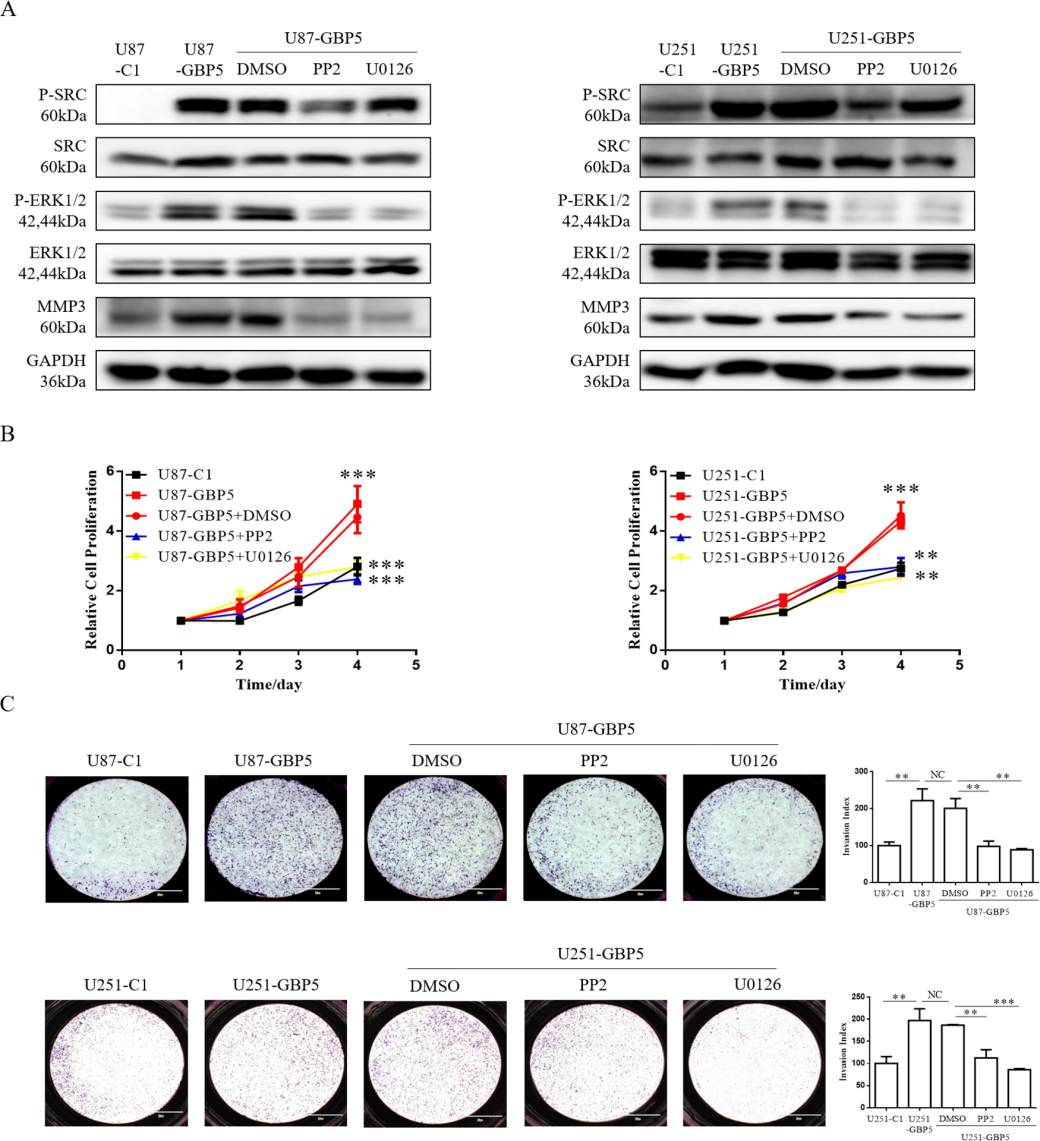
Src/ERK regulates GBP5-promoted MMP3 expression, cell proliferation and invasion. **A** Immunoblot analysis of p-Src, p-ERK1/2, and MMP3 in U87-C1, U87-GBP5, U251-C1, and U251-GBP5 cells. GAPDH was used as loading control. GBP5-expressing U87 and U251 cells were separately treated with pharmacological inhibitors of Src (PP2), ERK1/2 (U0126), and dimethyl sulfoxide (DMSO) vehicle for 8 h. **B** Effect of the blockade of Src or ERK1/2 with the inhibitors on the proliferation of U87 and U251 cells. **p < 0.01; ***p < 0.001. *n* = 3. **C** Effect of the blockade of Src or ERK1/2 with the inhibitors on the invasion of U87 and U251 cells. Invasion index = OD570 of invaded cells in treatment groups/OD570 of invaded cells in control groups. **p < 0.01; ***p < 0.001. NC = not statistically significant. *n* = 3.

We then asked whether Src and ERK1/2 were involved in GBP5-induced MMP3 expression, cell proliferation, and invasion. Pharmacological inhibitors of Src (PP2), ERK1/2 (U0126), and dimethyl sulfoxide (DMSO) vehicle were separately utilized to treat GBP5-expressing U87 and U251 cells for 8 h. Western blot showed that inhibition of Src phosphorylation partially decreased ERK1/2 phosphorylation level and GBP5-promoted MMP3 expression (Fig. 4A), and inhibition of ERK1/2 phosphorylation partially decreased MMP3 expression in cells without affecting p-Src levels. Importantly, cell proliferation and invasion analysis showed that blockade of Src or ERK1/2 with the inhibitors significantly reduced GBP5-promoted cell proliferation and invasion in U87 and U251 cells (Fig. 4B, C), suggesting that GBP5 promoted GBM aggressiveness via the Src/ERK1/2 MAPK/MMP3 pathway.

To further verify the above results, a patient-derived glioblastoma stem-like cell (GSC) GSC206 was chose to silence GBP5 expression by lentiviral shRNA (Supplementary Fig. S2A). Functional studies showed that knockdown of GPB5 slightly decreased cell proliferation (Supplementary Fig. S2B) and remarkably reduced cell migration and invasion (Supplementary Fig. S2C, D). Moreover, we observed that GBP5 silencing downregulated the levels of p-Src and p-ERK1/2 as well as protein levels of MMP3 (Supplementary Fig. S2E). Taken together, we believed that Src/ERK1/2 axis is involved in MMP3 expression, cell proliferation and invasion promoted by GBP5.

### GBP5 expression promotes glioblastoma growth and invasion in mice

**Fig. 5:**
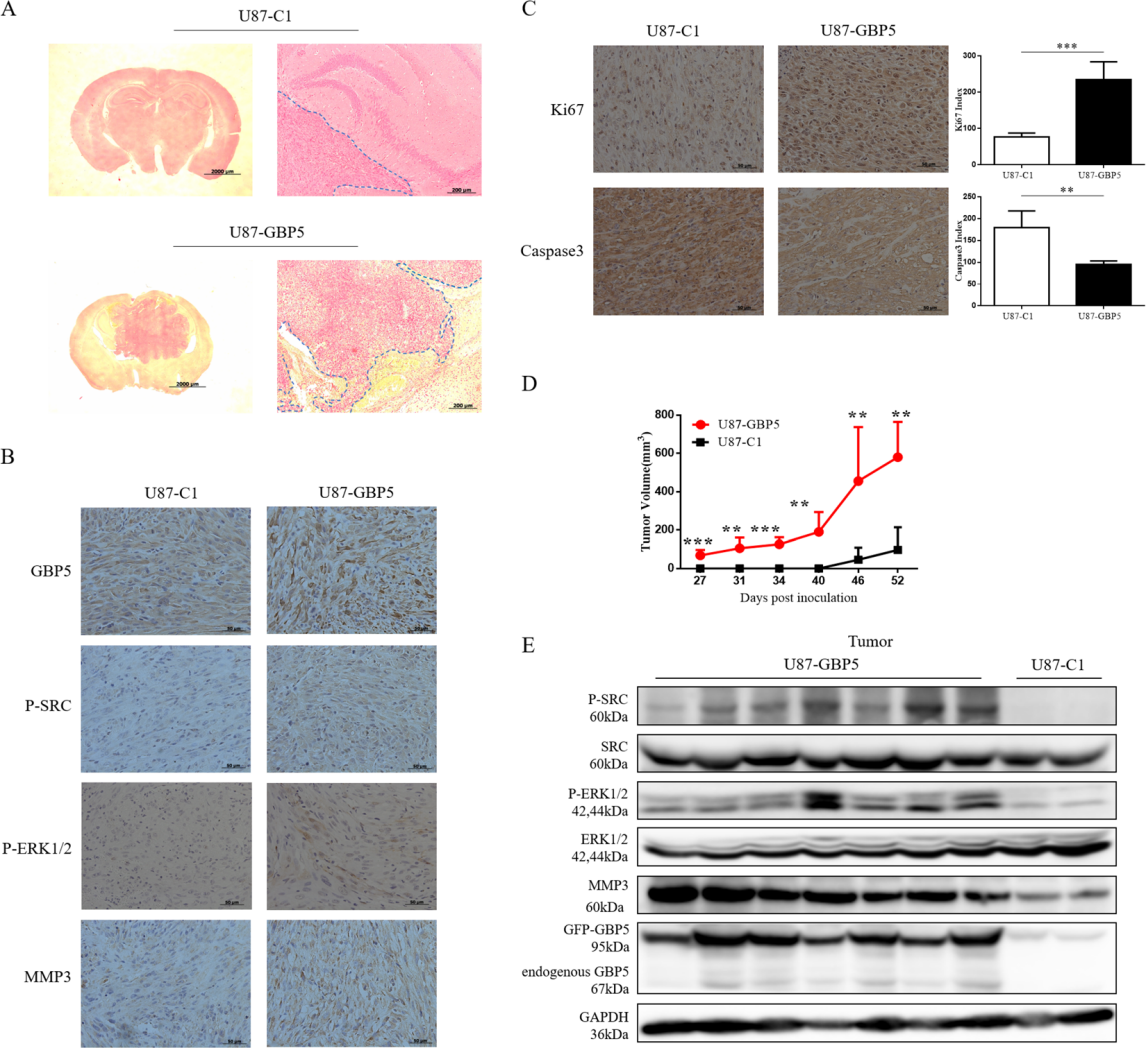
GBP5 expression promotes glioblastoma growth and invasion in mice. **A** H&E staining of brain sections of U87-C1 (control) and U87-GBP5 tumors. **B** IHC staining of GBP5, p-Src, p-ERK1/2, and MMP3 in U87-GBP5 and U87-C1 tumors. **C** IHC staining of Ki67 and Caspase 3 in U87-GBP5 and U87-C1 tumors. **p < 0.01; ***p < 0.001. *n* = 4. **D** Subcutaneous xenograft model studied the effects of GBP5 on GBM tumor growth in vivo. **p < 0.01; ***p < 0.001. *n* = 7. **E** Immunoblot analysis of p-Src, p-ERK1/2, and MMP3 in subcutaneous tumor tissues.

As mentioned above, GBP5 would enhance GBM cell proliferation and invasion in vitro. Therefore, we studied whether GBP5 had the same effect in vivo. U87-C1 cells (control group) and U87-GBP5 cells were stereotactically implanted into the brains of immunodeficient mice. The mice were euthanized after 26 days post inoculation, and the brains were collected followed by processing for pathological examinations with H&E staining. We observed larger and more invasive tumors in the brains of mice implanted with U87-GBP5 cells, while mice implanted with U87-C1 cells developed smaller tumors with smooth margins (Fig. 5A). Consistent with the results of previous in vitro studies, IHC staining of tumor sections showed higher levels of GBP5, p-Src, p-ERK1/2, and MMP3 in U87-GBP5 tumors relative to the control tumors (Fig. 5B). In addition, we observed more Ki67-positive cells and less Caspase 3-positive cells in the U87-GBP5 tumor compared to the control tumors, and the differences were 2.5–3 folds (Fig. 5C).

We also used the subcutaneous xenograft model in athymic nude mice to study the effects of GBP5 on GBM tumor growth in vivo. U87-C1 and U87-GBP5 cells were subcutaneously injected into the nude mice. The tumor growth curve showed that GBP5 expression significantly promoted the growth of GBM cells during the period of 52 days in vivo (Fig. 5D). As expected, Western blot analysis of these tumor tissues showed that overexpression of GBP5 increased the levels of p-Src, p-ERK1/2, and MMP3 (Fig. 5E).

**Fig. 6:**
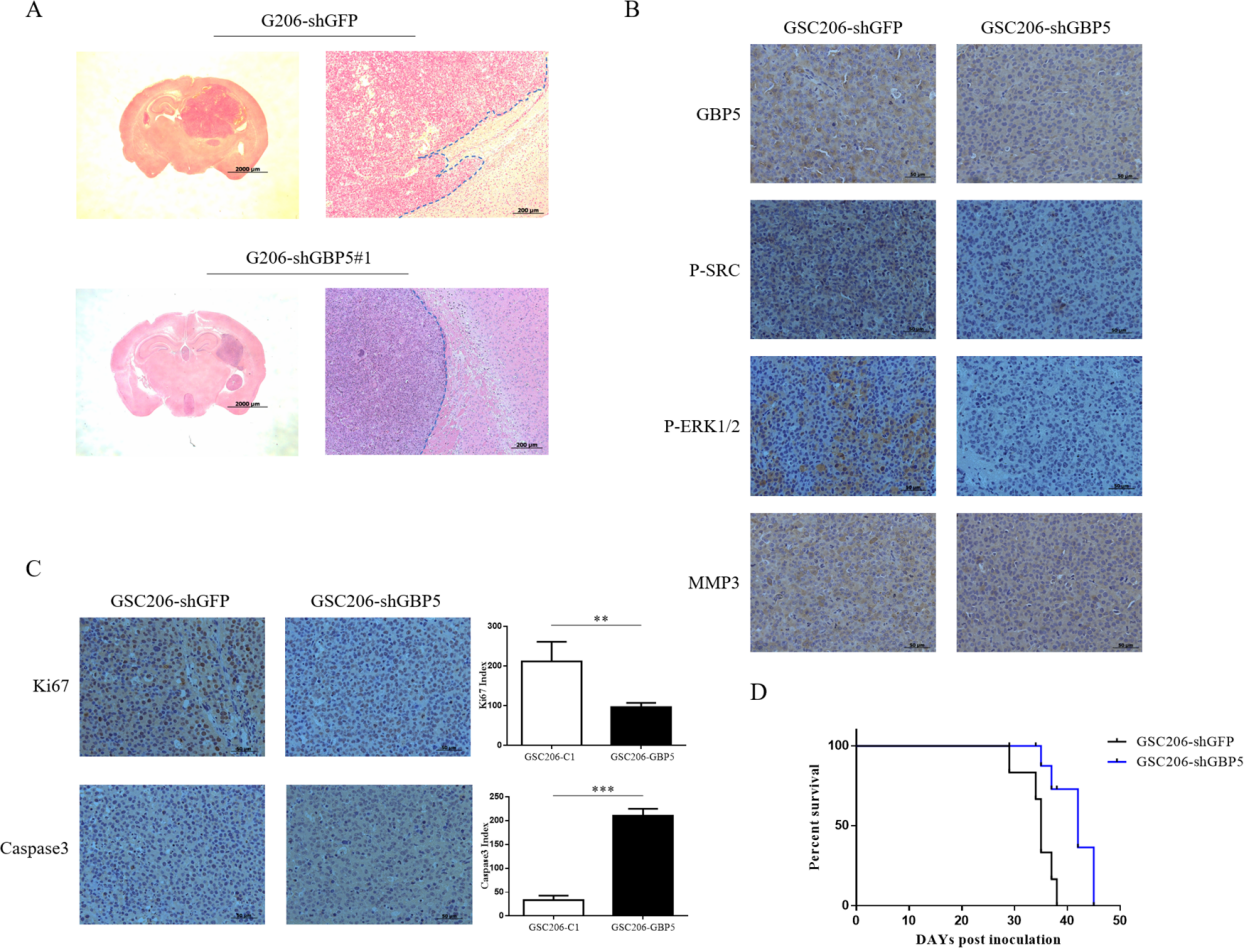
GBP5 knockdown inhibits glioblastoma growth and invasion in mice. **A** H&E staining of GSC206-shGBP5 and GSC206-shGFP (control) brain sections. **B** IHC staining of GBP5, p-Src, p-ERK1/2, and MMP3 in GSC206-shGBP5 and GSC206-shGFP tumors. **C** IHC staining of Ki67 and Caspase 3 in GSC206-shGBP5 and GSC-shGFP tumors. **p < 0.01; ***p < 0.001. *n* = 4. **D** Effect of GBP5 knockdown in the survival time of mice bearing tumor by survival studies. *n* = 6. *p* = 0.0057.

GBM stem-like cells, GSC206-shGBP5 and GSC206-shGFP (control group), were also used to study the effect of GBP5 expression on intracranial tumor growth and invasion. We found that GBP5 silencing significantly suppressed tumor growth and invasion (Fig. 6A). Consistently, the deletion of GBP5 reduced the expression of p-Src, p-ERK1/2, and MMP3 in tumors (Fig. 6B), and less cell proliferation and more cell apoptosis were observed by Ki67 and Caspase 3 staining (Fig. 6C). Importantly, survival studies showed that knockdown of GBP5 significantly prolonged the survival time of mice bearing tumor (35 vs. 45 days, *p* = 0.0057. Fig. 6D). In summary, the GBP5/Src/ERK1/2/MMP3 axis plays an important role in the cell proliferation of GBM, and the expression level of GBP5 may predict the prognosis of patients with GBM.

## Discussion

In this study, we investigated the biological role of GBP5 in malignant gliomas. We found that the full-length form GBP5a/b is highly expressed in GBM tumors and cell lines, and its expression is negatively correlated with the prognosis of the patients. Functionally, overexpression of GBP5 promotes the proliferation, migration, and invasion of GBM cells in vitro, and its expression significantly enhances the growth and invasion of GBM in vivo. We further demonstrated that the Src/ERK/MMP3 axis plays a role in GBP5-promoted GBM malignancy. These results suggest that GBP5 contributes to GBM tumor aggressiveness and may predict poor prognosis of GBM patients.

GBPs that can be induced by interferon^13,14^ constitute a family of seven members in humans, designated hGBP-1 to hGBP-7^15^. Best known for their protective immunity against microbial and viral pathogens, GBPs are poorly characterized in the development of several tumor types. For instance, GBP1 increase chemoresistance in ovarian and invasion in GBM^16,17^. Its high expression levels predict poor prognosis in head and neck cancer and promote lymph node metastasis in esophageal cancer^18,19^. Although previous studies have reported that GBP1 and GBP5 assemble to form homodimers and homotetramers^20^, the role of GBP5 in cancer remains largely unknown. Our previous studies demonstrated that GBP1 was required for EGFR-mediated matrix metalloproteinase 1 (MMP1) expression and invasion in GBM^17^. Unlike GBP1, GBP2-promoted glioma cell invasion mainly through induction of FN1^21^. GBP3 contributes to the glioma cell proliferation via regulating SQSTM1-ERK1/2 pathway^22^. Like other well-known members of the GBP family, GBP5 is also elevated in GBM, which predicts a poor survival. In the current study, we characterized the biological function of GBP5 in GBM. Interestingly, we noticed that GBP5 expression affected GBM cell proliferation both in vitro and in vivo, which is similar to the function of GBP3^22^. In contrast, the expression of GBP1 or GBP2 had minor effect on GBM cell growth in vitro but dramatically induced tumor growth in vivo^17,21^, indicating these GBPs may modulate tumor microenvironment. These results suggest that the biological function of each GBP member is not identical, although they are similar at the amino acid level. Moreover, they utilize different signaling pathways and effector genes to execute their biological functions, i.e., MMP1 is involved in GBP1-mediated cell invasion, while FN1 plays a role in GBP2-promoted cell invasion. The current study identified MMP3 was essential for GBP5-driven cell malignancy.

The current study focused on the full-length form of human GBP5a/b. Like human GBP5, murine GBP5 also has two splicing variants^23^. MuGBP5a is N-terminally truncated by 112 amino acids and C-terminally extended which lacks the isoprenylation motif. Thus, both human and murine GBP5 appear in different splicing variants, of which one has lost its isoprenylation site, suggesting the different isoforms may function differently. Although our data shows the full-length hGBP5a/b is the major form in GBM, but the short form of hGBP5ta is detectable in some GBM tissues and cells (Fig. 1A,B). Future work is needed to compare the specific function of each isoform in GBM.

Glioma cell invasion, radioresistance, and chemoresistance share similar signaling mediators, including Src family kinase and Mitogen-activated kinases (MAPKs). MAPKs (e.g., MEK, ERK, JNK, and p38) signaling results from upstream tyrosine kinases—Src family kinases (e.g., Src, Lyn, and Fyn)—either through activating point mutations or overexpression^24^. MAPKs induce ongoing signaling, thereby favoring cell cycle progression and the expression of antiapoptotic genes via the transcription factors^25^.

Matrix metallopeptidases (MMPs) is a family of structurally conserved zinc-dependent endopeptidases produced by tumor cells, playing an important role in central nervous system tumor growth and invasion^9^. MMPs participate in the degradation of extra cellular matrix macromolecules such as angiogenesis and cytokine activation, being responsible for tumor growth, invasion, metastasis, and angiogenesis^26–28^. There has been some evidence suggesting the importance of MMP3 in the pathogenesis of brain gliomas. For instance, elevated expression of MMP3 has been detected in human astrocytoma, especially in invasive glioma cells. Increased cytoplasmic MMP3 expression stimulates glioma cell invasiveness and migration^29–31^, making MMP3 a promising therapeutic target. The current study showed that MMP3 could be induced by GBP5 at both mRNA and protein levels in GBM cells and was essential for GBP5-driven impacts. Likewise, inhibition of the ERK1/2 signaling using the pharmacological inhibitors U0126 in cells can inhibit MMP3 production and action in an ERK1/2-dependent manner. Consistent with this, PP2-Src inhibitor-can inhibit the phosphorylation level of ERK1/2. Future studies are needed to identify transcriptional factor(s) essential for MMP3 induction in GBP5-expressing cells.

In conclusion, we demonstrated that GBP5 is highly elevated in GBMs and its expression may represent a prognostic risk factor in GBM. GBP5 activates the Src/ERK1/2 MAPK pathway to induce MMP3 expression which plays a key role in GBM growth and invasion. These findings suggest that GBP5 may serve as a potential target for GBM therapy.

## Methods

### Clinical samples

Human clinical specimens were obtained with a written approval of patients at the time of glioma surgery at the Second Affiliated Hospital of Soochow University. Tumor specimens were stored in liquid nitrogen and lysed in RIPA buffer for Western blot.

### Plasmids and reagents

The pEGFP-C1-GBP5 construct was provided by Dr. John MacMicking (Yale University). The human GBP5 gene was cloned at the SalI-BamHI cut sites of the pEGFP-C1 vector. Src family inhibitor PP2 and ERK1/2 inhibitor U0126 were purchased from Selleck Chemicals. pLKO.1-shGFP (CAAGCTGACCCTGAAGTTCAT) and pLKO.1-GBP5 (GCTCGGCTTTACTTAAGGATA) lentiviral shRNA plasmid were obtained from Sigma-Aldrich.

### Cell culture and gene transfection

GBM cell lines (U87, U251, DBTRG, A172, U373, LN229, SNB10, and LN428) and immortalized NHA and IPHFA were provided by Dr. Frank Furnari. All the cells were cultured in DMEM with 10% fetal bovine serum (FBS). GBM stem-like cell line GSC206 was originally derived from human primary GBM tissue and was cultured in DMEM/F-12 medium containing glutamine, B27, heparin (5 ng/ml), EGF (20 ng/ml), and FGF (20 ng/ml). According to the manufacturer’s instructions, pEGFP-C1 or pEGFP-GBP5 was transfected into U87 and U251 cells with lipofectamine 3000 reagent (Invitrogen). The cell lines for major studies were authenticated by STR profiling and tested for mycoplasma contamination.

The transfected U87-C1, U87-GBP5, U251-C1, and U251-GBP5 cells were selected with 4 mg/ml of G418 (Invitrogen), which were then subjected to fluorescence-activated cell sorting (FACS) on a BD FACSMelody™ Cell Sorter. And G418 (0.4 mg/ml) was used to maintain the positive cells.

### Virus production and infection

Lentiviral vector pLKO.1-shGFP or pLKO.1-shGBP5 and helper vectors pCMVDR8.91 and pMD.G-VSV-G were transfected into 293FT cells by Lipofectamine 3000 reagent. Twenty-four hours later, the medium was changed with fresh DMEM/10% FBS. The supernatants were collected every 24 h for 2 days and filtered with 0.45 μm nitrocellulose filter. Various supernatants were used to infect glioma cells for 24 h, and then selected with 2 μg/ml puromycin for 2 weeks. The stable pooled clones were verified by western blot.

### Western blot

Cells were washed twice with ice-cold PBS and then lysed in RIPA buffer (Beyotime Biotechnology) mixed with PMSF (100×), protease inhibitor cocktail (100×, Cell Signaling Technology), and PhosSTOPEASYpack (Roche). The reaction mixture was centrifuged at 12,000 × *g* for 15 min to remove cell fragments. Primary antibodies used were GBP5 Polyclonal Antibody (Proteintech, 13220-1), MMP3 Antibody (Proteintech, 17873-1), Phospho-Src Antibody (Cell Signaling Technology, 2105S), Src Antibody (Cell Signaling Technology, 2108S), Phospho-p44/42 MAPK (Erk1/2) Antibody (Cell Signaling Technology, 4370S), p44/42 MAPK (Erk1/2) Antibody (Cell Signaling Technology, 4695S), and GAPDH Antibody (Proteintech, 60004-1).

### Cell proliferation assay

Cell viability was assessed using a Cell Counting kit-8 (CCK-8; Dojindo, Tokyo, Japan). Briefly, the cells were plated into 96-well plates at a density of 1 × 10^3^ cells/100 μl/well and cultured for indicated days. 10 µl of CCK-8 reagent was added per well and incubated for 2 h at 37 °C prior to measuring the absorbance at 450 nm using a microplate reader (Synergy HTX, Biotek).

### Wound healing assay

The ability of cell migration was analyzed by wound healing assay. The same amount of U87-C1, U87-GBP5, U251-C1, or U251-GBP5 was seeded in the six-well cell culture plates. When the cells reached 80% confluence, the wounds were made by the 200 μl sterile pipette tips. After scratching, the isolated cells were washed gently by PBS and cultured in serum-free medium. The distance of cell migration was observed at four different spots, and the images of 0- and 24 h were taken at the same location. Cell migration index = migrated distance (at x h)/scratching distance (at 0 h).

### Invasion assay

In the invasion assay, Matrigel (Corning) was paved in the transwell chamber (8 μm pore size) at 4 °C overnight. Cells were diluted in serum-free DMEM in the upper chamber with a density of 2.5 × 10^4^ cells per insert. DMEM containing 10% FBS was added into the lower chamber. The remaining cells in the upper chamber were removed gently with cotton swabs after 24 h of culture, and the invaded cells on the bottom surface were fixed with 4% paraformaldehyde and then stained with 0.1% crystal violet for 20 min, finally washed for 3 times. After taking pictures with the microscope (Zeiss Axio Scope A1), the membrane was removed and dissolved in 5% SDS for 20 min and read by spectrophotometer (TECAN Infinite 200 Pro) for OD570. The invasion index was shown as 100% × OD570 of treated groups/OD570 of control groups.

### Real-time PCR

RT-qPCR was used to examine the mRNA levels of each gene expression. Total RNA was extracted by Trizol reagent (Invitrogen) and transformed into cDNA by Reverse Transcription Kit (Takara). qPCR was performed on an iCycler IQ using IQ Syber Green (Bio-Rad Laboratories) with diluted cDNA. All reactions were repeated at least three times in triplicate. Each sample was relatively quantified and normalized with GAPDH expression for comparison. Primers were listed in Supplementary Table 1.

### siRNA transfection

The MMP3 siRNA(h), GBP5 siRNA, and control siRNA were purchased from Santa Cruz Biotechnology. According to the product specification, lipofectamineRNAiMAX (Invitrogen) were used to transfect cells.

### Xenograft model and histological analysis

All animal experiments are in line with the ethical principles and guidelines approved by Soochow University Animal Care and Use Committee. 6–8 mice/group were randomly divided into groups and used for the animal studies. 5 × 10^6^ U87-C1 or U87-GBP5 cells were diluted with 100 μl PBS, and then subcutaneously injected into 5–6-week-old athymic nude female mice. The size of the tumors was measured with caliper regularly after tumor formation. The tumor volume was calculated according to the formula *V* = [1/2] *ab*^2^ (*a* and *b* presents the long and short diameters of the tumor). For the intracranial model, 1 × 10^6^ U87-C1/-GBP5 or GSC206-shGFP/-shGBP5 cells were diluted in 3 μl PBS and injected with stereotaxic instrument at 2 mm anterior and 1.5 mm lateral of the right hemisphere relative to bregma at a depth of 3 mm of the 5–6-week-old athymic nude mice. After 26 days, the nude mice were euthanized with ketamine and xylazine. The brains were paraffin-embedded, and the paraffin tissue blocks were cut into sections and stained with H&E. IHC staining was performed with antibodies against GBP5 (Proteintech), p-Src (Cell Signaling Technology), p-ERK1/2 (Cell Signaling Technology), MMP3 (Cell Signaling Technology), Caspase 3 (Proteintech), and Ki67 (Proteintech). Under microscopy, randomly selected ×400 high-power fields to count the Ki67-positive or Caspase 3-positive cells and calculated according to the formula: Number of Ki67- or Caspase 3-positive cells/total cell count × 100%.

### Statistical analysis

SPSS 20.0 (IBM SPSS, Armonk, NY, USA) software was used to conduct statistical analysis and data were presented as the mean ± standard deviation. Differences among groups were analyzed by Student’s *t* test, and multiple treatment groups within individual experiments were compared by ANOVA method. For survival curves related to allograft studies, *p* values were analyzed using the log-rank (Mantel-Cox) test. *P* < 0.05 was considered statistically significant. Sample sizes of all experiments were predetermined by calculations derived from our experience. No sample was excluded from the analyses. Animals were randomly grouped during our studies, and the data analysis was single masked. Investigators were not blinded to the group allocation during the experiment and outcome assessment. All the experiments were performed at least three times. The number of replicates was indicated in each figure legend.

## Supplementary information

Supplemental information and figures

## Data Availability

The expression profile of GBP5 and its relationship with GBM prognosis were generated or analyzed with public TCGA datasets in the current study. All other data generated or analyzed during this study are included in this published article (and its Supplementary Information Files).
