# Supplementary material for: GBP5 drives malignancy of glioblastoma via the Src/ERK1/2/MMP3 pathway
Source: Cell Death Dis. 2021 Feb 19;12(2):203. doi: 10.1038/s41419-021-03492-3 (PMC7896088; doi:10.1038/s41419-021-03492-3)
Supplement: Supplementary file 1 — Supplemental information and figures [file 41419_2021_3492_MOESM1_ESM.docx]

**Supplementary information**

**Supplemental Figure S1. Effect of GBP5 on the expression levels of the metastasis-related genes.** Relative mRNA levels of MMP3, MMP14, IL8, FN1 and IL6 genes were measured by RT-qPCR in U87-C1, U87-GBP5, U251-C1, U251-GBP5, SNB19-shGFP, and SNB19-shGBP5 cells. *, p<0.05; **, p<0.01; ***, p<0.001. NC, not statistically significant.

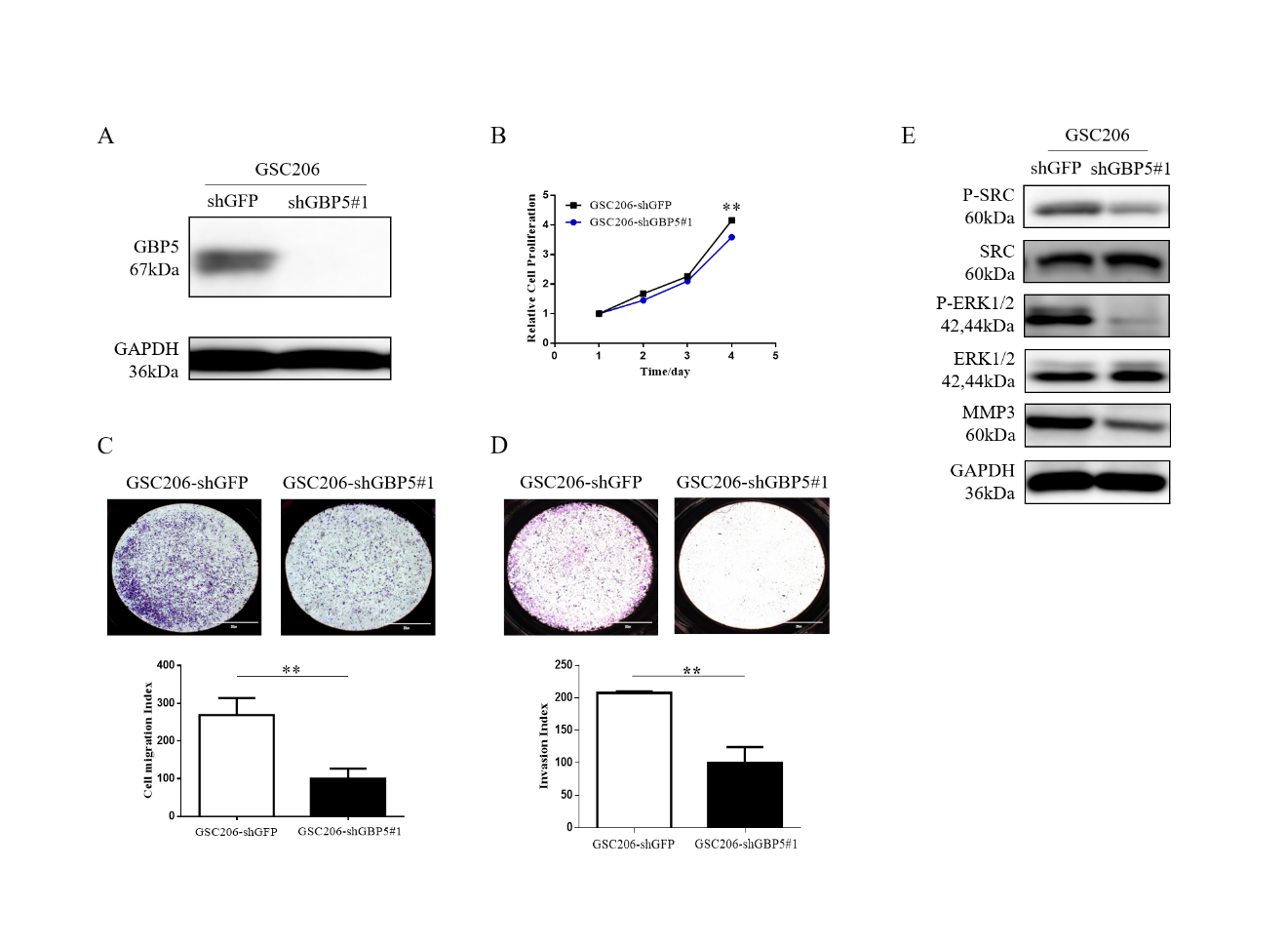
**Supplemental Figure S2. GBP5 knockdown inhibited GSC206 cell proliferation, migration, and invasion *in vitro*. (A).** Immunoblot analysis of GBP5 in GSC206-shGFP and GSC206-shGBP5 cells. GAPDH was used as loading control. **(B).** Effect of GBP5 knockdown on the proliferation of GSC206 cells. **, p<0.01. **(C).** Effect of GBP5 knockdown on GSC206 cell migration assessed by Transwell assay. Cell migration index= OD570 of invaded cells in treatment groups/OD570 of invaded cells in control groups. **, p<0.01. **(C).** Matrigel Transwell assay was done to examine the effect of GBP5 silencing on GSC206 cell invasion assessed by the matrigel invasion assay. Invasion index=OD570 of invaded cells in treatment groups/OD570 of invaded cells in control groups. **, p<0.01. **(D).** Immunoblot analysis of p-Src, P-ERK1/2, and MMP3 levels in GSC206-shGFP and GSC206-shGBP5 cells. GAPDH was used as loading control.

**Supplemental Table 1. List of primer sequences for RT-qPCR.**

| qPCR primer pairs | | |
| --- | --- | --- |
| GAPDH | F: 5'-GAAGGTGAAGGTCGGAGTCA-3' | R: 5'-TTGAGGTCAATGAAGGGGTC-3' |
| MMP3 | F: 5'-CACTCACAGACCTGACTCGG-3' | R: 5'-GAGTCAGGGGGAGGTCCATAG-3' |
| MMP14 | F: 5'-GGCGAGTATGCCACATACGA-3' | R: 5'-ACCCTGACTCACCCCCATAA-3' |
| FN1 | F: 5'-AGCAGACCCAGCTTAGAGTT-3' | R: 5'-GCAGAAGTGTTTGGGTGACT-3' |
| IL-6 | F: 5'-AGTGAGGAACAAGCCAGAGC-3' | R: 5'-GTCAGGGGTGGTTATTGCAT-3' |
| IL-8 | F: 5'-CCTGATTTCTGCAGCTCTGT-3 | R: 5'-AAATTTGGGGTGGAAAGGTT-3' |
